# An Update Overview on Brain Imaging Studies of Internet Gaming Disorder

**DOI:** 10.3389/fpsyt.2017.00185

**Published:** 2017-09-29

**Authors:** Aviv M. Weinstein

**Affiliations:** ^1^Department of Behavioral Science, Ariel University, Ariel, Israel

**Keywords:** Internet gaming disorder, brain imaging, functional magnetic resonance imaging, dopamine, reward

## Abstract

There are a growing number of studies on structural and functional brain mechanisms underlying Internet gaming disorder (IGD). Recent functional magnetic resonance imaging studies showed that IGD adolescents and adults had reduced gray matter volume in regions associated with attention motor coordination executive function and perception. Adolescents with IGD showed lower white matter (WM) integrity measures in several brain regions that are involved in decision-making, behavioral inhibition, and emotional regulation. IGD adolescents had also disruption in the functional connectivity in areas responsible for learning memory and executive function, processing of auditory, visual, and somatosensory stimuli and relay of sensory and motor signals. IGD adolescents also had decreased functional connectivity of PFC-striatal circuits, increased risk-taking choices, and impaired ability to control their impulses similar to other impulse control disorders. Recent studies indicated that altered executive control mechanisms in attention deficit hyperactivity disorder (ADHD) would be a predisposition for developing IGD. Finally, patients with IGD have also shown an increased functional connectivity of several executive control brain regions that may related to comorbidity with ADHD and depression. The behavioral addiction model argues that IGD shows the features of excessive use despite adverse consequences, withdrawal phenomena, and tolerance that characterize substance use disorders. The evidence supports the behavioral addiction model of IGD by showing structural and functional changes in the mechanisms of reward and craving (but not withdrawal) in IGD. Future studies need to investigate WM density and functional connectivity in IGD in order to validate these findings. Furthermore, more research is required about the similarity in neurochemical and neurocognitive brain circuits in IGD and comorbid conditions such as ADHD and depression.

## Introduction

### The Diagnosis and Brain Imaging of Internet Gaming Disorder (IGD)

Internet gaming disorder involves excessive or poorly controlled preoccupations, urges, or behaviors regarding computer and videogame play that lead to impairment or distress (1). The behavioral addiction model argues that IGD shows the features of excessive use despite adverse consequences, withdrawal phenomena, and tolerance that characterize substance use disorders. There is a debate whether IGD is the best clinical term for diagnosing Internet addiction, for example, Young argued that IGD is a loss of control over gaming (2, 3) and others have suggested that it is an impulse control disorder (4) or a part of the obsessive-compulsive disorder (5). In the fifth edition of the Diagnostic and Statistical Manual of Mental Disorders (6), IGD is identified in Section “Brain Activation” as a condition warranting further clinical research and experience before it might be considered for inclusion as a formal disorder. Previous reviews have described brain-imaging studies in IGD (7–12). In view of the rapid developments in brain research in IGD, particularly in adolescents, this review will summarize these studies and it will describe the gaps in our knowledge on brain imaging of IGD and bring them up to date to April 2017.

In PubMed, a search was conducted using the search terms “Internet addiction,” “Internet Gaming Disorder,” and “Pathological Internet use,” each of which was combined with each of the terms “brain imaging,” or “fMRI” or “PET” or “resting state” or “qualitative EEG” using the conjunction “AND.” Each term was required to be present in the “Title/Abstract” of the article. The search was further limited by “English” as the publication language and Publication Date from 2008 to April 2017. The only studies that were selected for the review were original research articles that were published in peer-reviewed journals. The search has yielded eligible 98 studies of which 76 were selected including 23 studies of the resting state, 18 studies of functional connectivity, 27 activation studies, and 8 studies of pharmacology. As a general caution, throughout this review, in making group comparisons, there are reported differences between IGD group and control groups but these differences do not imply a causal role of IGD. Group differences may reflect predisposing factors rather than decreases due to IGD.

### Brain Imaging Studies of the Resting State in IGD

Excessive Internet game use was associated with abnormal resting state activity in the brain regions that are responsible for impulse control, reward processing, and somatic representation of previous experiences (13). Adolescents with IGD also showed higher global cerebral blood flow in areas that are important for learning and memory (amygdala/hippocampus), conscious urges to use drugs (insula) executive function and inhibition (14). Individuals with IGD showed enhanced regional homogeneity (ReHo) in brain regions that relate with sensory-motor coordination (15, 16) and decreased ReHo in brain regions that are responsible for visual and auditory functions (15). The synchronization among these regions and the frontal lobe supports the evidence for enhancement of reward pathways (17). Both IGD and alcohol use disorder (AUD) patients had increased ReHo in the posterior cingulate cortex (PCC) an area associated with attention, future plans, and retrieval of autobiographical memories, whereas only IGD patients had decreased ReHo in the superior temporal gyrus an area associated with auditory processing and language (18). Scores on Internet addiction severity positively correlated with ReHo in the medial frontal cortex, precuneus/PCC, and left inferior temporal cortex (ITC) among participants with IGD (18). A further clarification as to the difference between IGD and AUD is provided by a recent study on resting-state quantitative electroencephalography (QEEG) patterns associated with IGD and AUD (19). The study showed that lower absolute beta power can be used as a potential trait marker of IGD whereas higher absolute power in the delta band may be a susceptibility marker for AUD. This study clarifies the unique characteristics of IGD as a behavioral addiction, which is distinct from AUD, by providing neurophysiological evidence. In conclusion, studies of the resting state provide preliminary evidence for cognitive function in IGD but apart from a single study (18) they cannot provide evidence as to the development of IGD. The structural changes to brain regions that are involved in the function and maintenance of IGD need further corroboration before any conclusions are drawn.

### Studies on the Brain’s Gray Matter Volume and White Matter (WM) Density

Early studies showed higher left striatal gray matter volume in IGD participants in functional magnetic resonance imaging (fMRI) and these measures negatively correlated with deliberation time on the Cambridge Gambling Task (20). This study has used a decision-making task that can help clarify the relationships between brain function, i.e., decision-making and structural changes in reward centers in the brain. Participants with IGD had also lower gray matter density (GMD) in areas involved in urges and the regulation of emotional behavior but no causality can be inferred from the results of this study (21). Progamers showed increased gray matter volumes of areas associated with attention and sensory-motor coordination (22). Studies also found lower WM density measures in several brain regions [orbitofrontal cortex (OFC), corpus callosum, cingulate, inferior frontal-occipital fasciculus, and corona radiation, internal, and external capsules] in adolescents with IGD (23). Participants with IGD also showed higher WM density in the thalamus and left PCC and higher WM density in the thalamus was associated with greater severity of IGD (24). Participants with IGD showed decreased gray matter volume in frontal brain regions and reduced WM in the parahippocampal gyrus and the limb of the internal capsule (25). This study showed an association between gray matter atrophy and WM density with length of time of play enabling to assess effects of play on the brain’s WM atrophy. Gray matter atrophy was reported in areas involved in cognitive and motor control and reduced WM density in areas involved in cognitive planning and control in IGD (26). Finally, IGD participants had lower GMD in brain regions that are involved in decision-making, behavioral inhibition and emotional regulation and reduced WM density in the inferior frontal gyrus, insula, amygdala, and anterior cingulate (27). In conclusion, these studies indicate preliminary findings of structural changes in gray matter volume and WM density in IGD. Regions consistently shown gray matter volume changes in IGD include the anterior cingulate, supplementary motor areas, cerebellum, insula, and the inferior temporal gyrus (12). There are few studies showing several brain regions that were associated with changes in WM density in IGD and therefore there is a need for studies that will select those regions that were repeatedly associated with structural changes in IGD. Except for a single study (25) that found an association between gray and WM changes and length of play, no inferences on causality can be drawn.

### Recent Studies in Young Adults and Adolescents

Recent studies showed that adolescents with IGD had lower diffusion measures in the areas are associated with attention and control, impulse control, motor function and emotional regulation (28). IGD adolescents also showed reduced gray matter volume in regions associated with attention motor coordination working memory and perception (29) findings that are compatible with studies on gray matter volume in IGD (21, 25, 26). Moreover, gray matter volume of the anterior cingulate cortex (ACC) negatively correlated with response errors on the Stroop task (29). IGD adolescents had reduced gray matter volume in prefrontal cortex and the amygdala that correlated with Barratt Impulsivity Scale hence enabling to make an association between function (impulsivity) and structure (gray matter in the OFC and the amygdala) (27). IGD participants also showed reduced WM density in the ACC and right dorsolateral–prefrontal cortex, regions associated with executive function such as the Stroop task (30). Increased videogame play was associated with delayed development of the OCF, pallidum, putamen, hippocampus, caudate/putamen insula, and the thalamus. Furthermore, higher mean diffusivity measures in the areas of the thalamus, hippocampus, putamen, and the insula was associated with lower intelligence (31). These measures indicate an association between videogame play, intelligence, and brain development but cannot enable any causal inferences. There is also evidence for reduced WM efficiency in the frontal cortex, ACC and pallidum in IGD (32). IGD subjects had also increased WM density and decreased diffusivity in frontal fiber tracts (33). In conclusion, the studies reviewed so far present structural changes in adolescents and young adults with IGD that require replication and validation. Furthermore, these are cross-sectional studies precluding any inference on causality.

See Table 1 for resting state and structural studies of Internet and gaming disorder.

**Table 1:** Resting state and structural studies of Internet and gaming disorder.^a^

Reference	Methods	Participants	Main findings and evaluations
Park et al.(13)	Regional cerebral metabolic rates of glucose (rCMRglu) in positron-emission tomography (PET)	Eleven Internet and gaming over users and nine control participants	Increased activity in the OFC, striatum, and sensory regions
			Evaluation—a cross-sectional study with a small number of participants

Liu et al.(16)	Regional homogeneity (ReHo) measure in MRI	Nineteen IGD college students (11 males 8 females) and 19 control participants	Enhanced ReHo in the cerebellum, brainstem, right cingulate gyrus, bilateral parahippocampus, right frontal lobe (rectal gyrus, inferior frontal gyrus and middle frontal gyrus), left superior frontal gyrus, left precuneus, right postcentral gyrus, right middle occipital gyrus, right inferior temporal gyrus, left superior temporal gyrus, and middle temporal gyrus
			Evaluation—a cross-sectional study—preliminary results

Kuhn et al.(20)	Gray matter volume measure in MRI	Seventy-six frequent compared with 78 infrequent adolescent video game players (14 years old)	Higher left striatal gray matter volume negatively correlated with deliberation time on Cambridge Gambling Task Activity on the Monetary Incentive Delay task was enhanced during feedback of loss compared with no loss
			Evaluation—a cross-sectional study enables to assess relationships between a cognitive task and brain’s GMD

Zhou et al.(21)	Gray matter volume measure in MRI	Eighteen Internet addicted adolescents (16 males 2 females) and 15 control participants (13 males)	Lower gray matter density (GMD) in the left ACC, left PCC, left insula, and left lingual gyrus
			Evaluation—a cross-sectional study—preliminary results of gray matter in IGD

Yuan et al.(25)	White matter (WM) fractional anisotropy (FA) changes using the diffusion tensor imaging (DTI) in MRI	Eighteen adolescents with IGD (12 males) and 18 control participants	Decreased gray matter volume in the bilateral DLPFC, the SMA, the OFC, the cerebellum and the left rostral ACC. Enhanced FA value of the left PLIC and reduced FA value in the WM within the right PHG
			Gray matter volumes of the DLPFC, rACC, SMA, and WM FA changes of the PLIC correlated with the duration of Internet addiction
			Evaluation—a cross-sectional study that enables evaluation of GM and WM changes over time of play

Dong et al.(12)	Regional homogeneity (ReHo) measure in MRI	Fifteen Internet and gaming disorder and 14 control participants	Enhanced regional homogeneity (ReHo) in the brainstem, inferior parietal lobule, left posterior cerebellum, and left middle frontal gyrus, decreased ReHo in temporal, occipital and parietal cortex
			Evaluation—a cross-sectional study—preliminary findings of ReHo

Han et al.(22)	Gray matter volume measure in MRI	Twenty IGD participants, 18 male control participants and 17 progammers	Increased impulsiveness and perseverative errors, and volume in left thalamus gray matter, but decreased gray matter volume in inferior temporal gyri, right middle occipital gyrus, and left inferior occipital gyrus
			Evaluation—a cross-sectional study preliminary findings of GM changes

Lin et al.(23)	Brain WM integrity measured by diffusion tensor imaging (DTI) in MRI. Whole brain voxel-wise analysis of fractional anisotropy (FA) was performed by tract-based spatial statistics (TBSS)	Seventeen Internet addiction disorder (14 males) and 16 control adolescents	Lower FA in the OFC, corpus callosum, cingulate, inferior frontal–occipital fasciculus, and corona radiation, internal and external capsules, FA values in the left genu of the corpus callosum negatively correlated with scores on the screen for child anxiety related emotional disorders, and between FA values in the left external capsule and Young’s Internet addiction scale
			Evaluation—a cross-sectional study enables assessment of WM changes in relation to Internet addiction and anxiety severity

Dong et al. (64)	WM integrity using diffusion tensor imaging (DTI) in MRI	Sixteen Internet gaming addicted participants and 15 control participants	Higher fractional anisotropy (FA), in the thalamus and left PCC. Higher FA in the thalamus was associated with greater severity of Internet addiction
			Evaluation—a cross-sectional study enables evaluation of changes in WM in relation to IGD severity

Weng et al. (26)	GMD and WM density changes using Voxel-based morphometry (VBM) analysis and tract-based spatial statistics (TBSS) was reported	Seventeen IGD participants (13 females and 4 males) and 17 control participants (15 females 2 males)	Gray matter atrophy in the right OFC, bilateral insula, and right supplementary motor area
			Reduced FA in the right genu of corpus callosum, bilateral frontal lobe WM, and right external capsule. Gray matter volumes of the right OFC, bilateral insula and FA values of the right external capsule positively correlated with Young’s Internet addiction scores
			Evaluation—a cross-sectional study that enables evaluation of GM changes in relation to IGD severity

Hong et al. (35)	Cortical thickness in MRI	Fifteen male adolescents diagnosed with Internet addiction and 15 male control participants	Decreased cortical thickness in the right lateral OFC
			Evaluation—a cross-sectional study with preliminary results of cortical thickness

Yuan et al. (34)	Cortical thickness in MRI	Eighteen adolescents with Internet gaming disorder and 18 control participants	Increased cortical thickness in the left precentral cortex, precuneus, inferior middle frontal cortex temporal and middle temporal cortices
			Decreased cortical thicknesses of the left lateral OFC, insula, lingual gyrus, the right postcentral gyrus, entorhinal cortex and inferior parietal cortex
			Cortical thicknesses of the left precentral cortex, precuneus, and lingual gyrus correlated with duration of online gaming addiction and the cortical thickness of the OCF correlated with the impaired task performance during the color-word Stroop task
			Evaluation—a cross-sectional study that enables the evaluation of the relationship between cortical thickness and duration of online gaming and also with cognitive performance

Sun et al. (28)	Diffusional kurtosis imaging (DKI) in the detection of gray matter diffusion	Eighteen participants with Internet gaming disorder and 21 control participants	Lower gray matter diffusion in the right anterolateral cerebellum, right inferior and superior temporal gyri, right SMA, middle occipital gyrus, right precuneus, postcentral gyrus, right inferior frontal gyrus, left lateral lingual gyrus, left paracentral lobule, left ACC, and median cingulate cortex, bilateral fusiform gyrus, insula, PCC, and thalamus
			Higher GM volume in the right inferior and middle temporal gyri, and right PHG, and lower volume in the left precentral gyrus
			Evaluation—a cross-sectional study that measures GM diffusion—preliminary findings

Son et al. (19)	Resting-state quantitative electroencephalography (QEEG)	Thirty-four participants with IGD, 17 with AUD, and 25 healthy control participants	IGD participants had lower absolute beta power than AUD and the healthy control group. The AUD group showed higher absolute delta power than IGD and the healthy control group. No significant correlations between the severity of IGD and QEEG activities in patients with IGD
			Evaluation—a cross-sectional study—enables evaluation of EEG in relation to IGD severity

Wang et al. (24)	Gray matter volume measure in MRI	Twenty-eight Internet participants with Internet gaming disorder and 28 control participants	Gray matter volume of the bilateral ACC, precuneus, SMA, SPL, left DLPFC, left insula, and bilateral cerebellum decreased in IGD participants compared with healthy control participants
			Gray matter volume of the ACC negatively correlated with the incongruent response errors on the Stroop
			Evaluation—a cross-sectional study that enabled assessment of relationship between GM changes with cognitive performance

Kim et al. (18)	Regional homogeneity (ReHo) measure in MRI	Sixteen patients with Internet gaming addiction (IGD), 14 alcohol use disorder (AUD), and 15 control participants	IGD and AUD participants had increased ReHo in the PCC. IGD participants showed decreased ReHo in the right superior temporal gyrus compared with AUD and control participants. Patients with AUD showed decreased ReHo in the ACC
			Scores on Internet addiction severity positively correlated with ReHo in the medial frontal cortex, precuneus/PCC, and left inferior temporal cortex (ITC) among participants with IGD
			Evaluation—a cross-sectional study that enabled a comparison of ReHo measures between IGD and AUD. The study enabled assessment of relationship between ReHo measures with IGD severity

Lin et al. (27)	GMD and WM density changes using voxel-based morphometric analysis in MRI	Thirty-five participants with Internet gaming disorder and 36 control participants	Lower GMD in the bilateral inferior frontal gyrus, left cingulate gyrus, insula, right precuneus, and right hippocampus. Lower WM density in the inferior frontal gyrus, insula, amygdala, and anterior cingulate
			Evaluation—a cross-sectional study with a large number of participants enables GM and WM analysis in IGD

Takeuchi et al. (32)	Diffusion tensor imaging mean diffusivity (MD)	A hundred and fourteen boys and 126 girls	The amount of videogame play was associated with increased MD in the left middle, inferior, and orbital frontal cortex; left pallidum; left putamen; left hippocampus; left caudate; right putamen; right insula; and thalamus in both cross-sectional and longitudinal analyses
			Higher MD in the areas of the left thalamus, left hippocampus, left putamen, left insula, and left Heschl gyrus was associated with lower intelligence
			Evaluation—a cross-sectional study with a very large sample enables cross-sectional and longitudinal assessment of diffusion in the brain

Yuan et al. (30)	White matter (WM) integrity and connectivity	Twenty-eight IGD adolescents and 25 control participants	Reduced FA in the ACC-right dorsolateral prefrontal cortex pathways in IGD
			Evaluation—a cross-sectional study assessing WM integrity

Zhai et al. (32)	WM integrity measured with diffusion tensor imaging (DTI)	Sixteen right-handed adolescents with IGD and 16 control participants	Reduced nodal efficiency in frontal cortex, ACC, and pallidium in IGD. The global efficiency of WM network correlated with the IAT scores in IGD
			Evaluation—a cross-sectional study assessing WM integrity and also enabled assessment of the relationships between WM changes and IGD severity

Jeong et al. (33)	WM integrity and connectivity	A hundred and eighty-one male patients including 58 of IGD subjects without psychiatric comorbidity and 26 male control subjects	Increased FA values within forceps minor right anterior thalamic radiation, right corticospinal tract, right inferior longitudinal fasciculus, right cingulum to hippocampus and right inferior fronto-occipital fasciculus (IFOF) decreases in RD value within forceps minor, right anterior thalamic radiation, and IFOF relative to control subjects
			Evaluation—a cross-sectional study assessing WM integrity and connectivity

Park et al. (92)	Qualitative EEG	Sixteen adolescent males with ADHD and IGD, 15 adolescent males with ADHD-only, and 15 healthy adolescent males	Compared to the ADHD-only group, the ADHD + IGD group showed lower relative delta power and greater relative beta power in temporal regions. The relative theta power in frontal regions was higher in ADHD-only group compared to HC group. Increased neuronal connectivity within the parieto-occipital and temporal regions for the ADHD + IGD group
			Evaluation—a cross-sectional study assessing qualitative EEG—low localization

Youh et al. (95)	Qualitative EEG	Fourteen males with MDD and IGD and 15 male with MDD-only	An association between decreased interhemispheric connectivity in the frontal region and vulnerability to attention problems in patients with MDD and IGD
			Interhemispheric and intrahemispheric coherence value for the alpha band was significantly lower in MDD + IGD than MDD-only patients. Intrahemispheric coherence values for the beta band were higher in MDD + IGD than MDD-only patients. Increased intrahemisphere connectivity in the frontal–temporal–parietal–occipital areas may result from excessive online gaming
			Evaluation—a cross-sectional study assessing qualitative EEG—low localization

*^a^Studies arranged chronologically*.

*DLPFC, dorsolateral prefrontal cortex; SMA, supplementary motor area; OFC, orbitofrontal cortex; ACC, anterior cingulate cortex; PLIC, posterior limb of the internal capsule; PHG, parahippocampal gyrus; PCC, posterior cingulate cortex; STG, superior temporal gyrus; MPFC, medial prefrontal cortex; AG, angular gyrus; SPL, superior parietal lobule*.

### Cortical Thickness

Studies that measured cortical thickness in fMRI revealed conflicting results of increased and decreased cortical thickness in several brain regions in adolescents with IGD (34, 35). The cortical thickness of the OCF correlated with impaired performance on the color-word Stroop task (35). The apparent contradiction between the two studies showing increased and decreased cortical thickness seems to suggest that the changes are not robust and merit further studies.

## Functional Connectivity

### Functional Connectivity at a Resting State

Early studies in participants with IGD showed increased functional connectivity between regions that are associated with cognitive regulation, signal processing, and storage of relevant auditory-verbal memory processes (36). These findings are consistent with current models emphasizing the role of cortical-subcortical pathology in addiction (37). Disruption in functional connectivity in IGD may also affect motivation and reward. Smokers with IGD exhibited decreased functional connectivity with brain regions that are involved in the evaluation and expectancy of reward (38). IGD participants showed reduced connectivity in areas responsible for executive function and increased connectivity in sensory-motor brain networks (39). Lower functional connectivity in IGD affected executive control networks (40). IGD participants also showed increased volume of the caudate and nucleus accumbens as well as reduced resting state functional connectivity of dorsal prefrontal cortex (DLPFC)-caudate and OCF and the nucleus accumbens, regions associated with reward (41). Impulsivity also correlated negatively with functional connectivity between the amygdala, dorsolateral prefrontal cortex, and the OCF (42) and it was associated with alterations over the frontal-limbic connections (43). In conclusion, these are few studies with several regions that have been specifically related to drug addiction but also others that are associated with general cognitive function so more studies need to be conducted in order to select related from unrelated brain regions.

### Recent Studies in Adolescents

Consistent with recent models emphasizing the role of cortical–subcortical pathology in addiction, adolescents with IGD showed reduced functional connectivity in cortical–subcortical circuits (44). IGD adolescents had also disruption in the functional connectivity in areas responsible for learning memory and executive function, processing of auditory, visual, and somatosensory stimuli and relay of sensory and motor signals (45). IGD adolescents showed decreased functional connectivity of PFC and striatal circuits areas associated with reward (46). Adolescents with IGD also showed reduced dorsal putamen functional connectivity with the posterior insula-parietal operculum (47). IGD participants had increased volumes of dorsal striatum (caudate) and ventral striatum (nucleus accumbens) (48). IGD participants also exhibited enhanced resting state functional connectivity between the anterior insula and areas that are involved in salience, craving, self-monitoring, and attention (49). Furthermore, IGD participants had stronger functional connectivity between left posterior insula and brain regions indicating reduced ability to inhibit motor responses and control over craving for Internet gaming (49). IGD participants had decreased connectivity measures between parts of the frontal cortex (50). Finally, IGD adolescents demonstrated increased functional connectivity in brain regions involved in working memory, spatial orientation and attention processing (51). In conclusion, participants with IGD showed reduced connectivity in several areas that are responsible for executive function, cognitive control, sensory processing motivation and reward. Some of these regions are common to IGD and substance use disorders but others are associated with general mechanisms of learning, memory and information processing that are not specific to IGD and substance use disorder, so a better selection is required and no inferences on causality can be drawn from present studies. See Table 2 for studies on functional connectivity in Internet and gaming disorder.

**Table 2:** Studies of functional connectivity in fMRI.^a^

Reference	Method	Participants	Main findings and evaluation
Ding et al.(36)	Functional connectivity in fMRI	Seventeen adolescents with Internet gaming disorder and 24 control adolescents	Increased functional connectivity in the bilateral cerebellum posterior lobe and middle temporal gyrus. Decreased connectivity in the bilateral inferior parietal lobule and right inferior temporal gyrus. Connectivity with the PCC positively correlated with Internet Addiction Scores in the right precuneus, PCC, thalamus, caudate, nucleus accumbens, SMA, and lingual gyrus. It negatively correlated with the right cerebellum, anterior lobe and left SPL
			Evaluation—a cross-sectional study assessing functional connectivity. Enables assessment of the relationship between connectivity and IGD severity

Hong et al.(47)	Functional connectivity in fMRI	Twelve adolescents with Internet addiction and 11 control participants	Reduced functional connectivity in corticosubcortical circuits (~24% with prefrontal and ~27% with parietal cortex). Bilateral putamen was the most extensively involved subcortical brain region
			Evaluation—a cross-sectional study assessing functional connectivity

Feng et al.(14)	Arterial spin-labeling (ASL) perfusion in fMRI	Fifteen adolescents with IGA and 18 control adolescents	Higher global cerebral blood flow (CBF) in the left inferior temporal lobe/fusiform gyrus, left PHG/amygdala, right medial frontal lobe/ACC, left and right insula, right middle temporal gyrus, right pre-central gyrus, left SMA, left cingulate gyrus, and right inferior parietal lobe. Lower CBF in the left middle temporal gyrus, left middle occipital gyrus, and right cingulate gyrus
			Evaluation—a cross-sectional study assessing perfusion. Preliminary findings

Wee et al. (45)	Functional connectivity in fMRI	Seventeen adolescents with IGD and 16 control participants	Disruption in the functional connectivity with the frontal, occipital, and parietal lobes
			Functional connectivity with the frontal, occipital, and parietal lobes correlated with the IAD severity
			Evaluation—a cross-sectional study assessing functional connectivity. Enables assessment of the relationship between connectivity and IGD severity

Chen et al. (38)	Functional connectivity in fMRI	Twenty-nine smokers with IGD, 22 non-smokers with IGD, and 30 control participants	Decreased resting state functional connectivity with posterior cingulate cortex in the right rectus gyrus. Increased resting state functional connectivity with the left middle frontal gyrus in smokers with IGA compared with non-smokers with IGA
			Evaluation—a cross-sectional study assessing functional connectivity. Enables assessment of the relationship between connectivity and IGD severity.

Dong et al. (40)	Functional connectivity in fMRI	Thirty-five IGD and 36 control participants	Lower functional connectivity in executive control networks. Functional connectivity measures in executive control networks were negatively correlated with Stroop effect and positively correlated with brain activations in executive-control regions across groups
			Evaluation—a cross-sectional study assessing functional connectivity. Enables assessment of the relationship between connectivity and cognitive function

Ko et al. (42)	GMD and functional connectivity in fMRI	Thirty males with IGD and 30 control participants	Lower GMD in the bilateral amygdala and higher impulsivity. Lower functional connectivity with the left amygdala over the left DLPFC and with the right amygdala over the left DLPFC and OFC. Higher functional connectivity with the bilateral amygdala over the contralateral insula
			The functional connectivity between the left amygdala and DLPFC negatively correlated with impulsivity. The functional connectivity of the right amygdala to the left DLPFC and OFC also negatively correlated with impulsivity
			Evaluation—a cross-sectional study assessing functional connectivity. Enables assessment of the relationship between connectivity and impulsivity

Hong et al. (47)	Functional connectivity in fMRI in subdivisions of striatum	Twelve male adolescents with Internet gaming disorder and 11 male control participants	Reduced dorsal putamen functional connectivity with the posterior insula-parietal operculum. Time spent playing online games predicted significantly greater functional connectivity between the dorsal putamen and bilateral primary somatosensory cortices
			Lower functional connectivity between the dorsal putamen and bilateral sensorimotor cortices in healthy control participants
			Evaluation—a cross-sectional study assessing functional connectivity. Enables assessment of the relationship between connectivity and time spent playing online

Wang et al. (50)	Functional connectivity and voxel-mirrored homotopic connectivity (VMHC) method	Seventeen participants with IGD and 24 healthy control participants	Decreased VMHC between the left and right superior frontal gyrus (orbital part), inferior frontal gyrus (orbital part), middle frontal gyrus, and superior frontal gyrus
			Evaluation—a cross-sectional study assessing functional connectivity

Zhang et al. (49)	Functional connectivity of the insula in fMRI	Seventy-four young adults with Internet gaming disorder (IGD) and 41 control participants	Enhanced functional connectivity between the anterior insula and a network of regions including ACC, putamen, angular gyrus, and precuneous. Stronger functional connectivity between the posterior insula and postcentral gyrus, pre-central gyrus, SMA, STG. IGD severity was positively associated with connectivity between the anterior insula and AG, and STG, and with connectivity between the posterior insula and STG. Duration of Internet gaming was positively associated with connectivity between the anterior insula and ACC
			Evaluation—a cross-sectional study assessing functional connectivity. Enables assessment of the relationship between connectivity and duration of Internet gaming

Cai et al. (48)	Functional connectivity in fMRI in striatal nuclei (caudate, putamen, and nucleus accumbens) volumes	Twenty-seven adolescents with IGD and 30 control participants	Increased volumes of dorsal striatum (caudate) and ventral striatum (nucleus accumbens) and more errors on the Stroop task. Caudate volume correlated with Stroop task performance and nucleus accumbens (NAc) volume was associated with the Internet addiction test (IAT) score in the IGD group
			Evaluation—a cross-sectional study assessing functional connectivity with the striatum. Enables assessment of the relationship between volume of the striatum with cognitive performance and IGD severity

Du et al. (51)	Functional connectivity density (rsFCD) in fMRI	Twenty-seven male IGD adolescents and 35 healthy control participants	IGD adolescents exhibited higher global/long-range rsFCD in the bilateral dorsal lateral prefrontal cortex (DLPFC) and the right inferior temporal cortex (ITC)/fusiform compared with healthy control participants
			Evaluation—a cross-sectional study assessing functional connectivity

Jin et al. (46)	Functional connectivity	Twenty-five adolescents with IGD and 21 age- and gender-matched control participants	Decreased functional connectivity between the insula, and temporal and occipital cortices and dorsal striatum, pallidum, and thalamus in IGD. Some of those changes were associated with the severity of IGD
			Evaluation—a cross-sectional study assessing functional connectivity. Enables assessment of the relationship between connectivity and IGD severity

Wang et al. (39)	Functional connectivity	Thirty-seven IGD subjects and 35 matched control subjects	Reduced connectivity in the prefrontal cortex, left posterior cingulate cortex, right amygdala, and bilateral lingual gyrus, and increased functional connectivity in sensory-motor-related brain networks in IGD
			Evaluation—a cross-sectional study assessing functional connectivity

Zhang et al. (49)	Functional connectivity of insula-based network	Seventy-four young adults with IGD and 41 age- and gender-matched control subjects	Enhanced functional connectivity between the anterior insula and the ACC, putamen, angular gyrus, and precuneous. Stronger functional connectivity between the posterior insula and postcentral gyrus, precentral gyrus, supplemental motor area, and superior temporal gyrus (STG). IGD severity was positively associated with connectivity between the anterior insula and angular gyrus, and STG, and with connectivity between the posterior insula and STG. Duration of Internet gaming was positively associated with connectivity between the anterior insula and ACC
			Evaluation—a cross-sectional study assessing functional connectivity. Enables assessment of the relationship between connectivity and duration of Internet gaming

Du et al. (51)	Functional connectivity	Twenty-seven male IGD adolescents and 35 control participants	Enhanced functional connectivity in the bilateral dorsal lateral prefrontal cortex (DLPFC) and the right inferior temporal cortex (ITC)/fusiform
			Evaluation—a cross-sectional study assessing functional connectivity

Park et al. (10, 43, 92)	Functional connectivity in fMRI	Nineteen Internet gaming disorder adolescents and 20 age-matched control participants	Higher impulsiveness and higher global efficiency and lower local efficiency pathological states. Topological alterations were specifically attributable to inter-regional connections incident on the frontal region, and the degree of impulsiveness was associated with the topological alterations over the frontal-limbic connections
			Evaluation—a cross-sectional study assessing functional connectivity

Yuan et al. (41)	Functional connectivity in fMRI	Twenty-eight IGD adolescents and 25 control participants	Reduced FA in salience network, right central executive network tracts, and between-network (the ACC-right DLPFC tracts). Correlation between the effective and structural connection from salience network to central executive network and the number of errors during incongruent condition in Stroop task in both IGD and control participants
			Evaluation—a cross-sectional study assessing functional connectivity. Enables assessment of the relationship between connectivity and cognitive performance

*^a^Studies arranged chronologically*.

*DLPFC, dorsolateral prefrontal cortex; SMA, supplementary motor area; OFC, orbitofrontal cortex; ACC, anterior cingulate cortex; PLIC, posterior limb of the internal capsule; PHG, parahippocampal gyrus; PCC, posterior cingulate cortex; STG, superior temporal gyrus; MPFC, medial prefrontal cortex; AG, angular gyrus; SPL, superior parietal lobule*.

## Brain Activation

### Cue-Exposure Activation Studies of Videogame Urges

Males with IGD had greater activation in the meso-cortico-limbic system compared with females while playing a space-infringement game (52). Several frontal striatal and limbic brain regions were activated in IGD participants in fMRI (53). A longitudinal study of cue-reactivity found activation in the ACC and OCF of IGD participants over 6 weeks in fMRI (54). Gaming cues also activated regions that are associated with urges to play games (55). Furthermore, Gaming and smoking cues shared similar mechanisms of cue-induced reactivity of the frontal-limbic network (56). Exposure to World of Warcraft game figures activated brain regions that were associated with cognitive, emotion and motivation-related function in IGD participants (57). IGD participants had increased activation in regions that are associated with visuospatial orientation, space, attention, mental imagery and executive function (58). IGD participants also showed attention bias to short presentations of game pictures and enhanced brain responses in the medial prefrontal cortex and the ACC (59). IGD adolescents showed activation of areas associated with visual–spatial attention and body self-awareness during ball-throwing animations simulating the experience of “disembodied state” in cyberspace (60, 61). In conclusion, several studies have shown a consistent pattern of brain regions that were activated in response to video playing stimuli in IGD. Secondly, studies that use tasks that simulate reward (15) enable to assess the effects of cue exposure on the brain. Finally, only a single brain-imaging study (54) followed cue-activation over time enabling an assessment of causality.

### Recent Activation Studies in IGD

Internet gaming disorder participants exhibited higher cue-induced activations within the ventral and dorsal striatum compared with healthy control participants (62). There was a positive correlation between dorsal striatum activation and duration of IGD indicating a transition from ventral to dorsal striatal processing among individuals with IGD (60). Second, Internet gaming addiction appears to be associated with increased identification with one’s avatar, indicated by high left Angular Gyrus activations in pathological Internet gamers (63). This experimental manipulation can suggest how self-identification during videogame play can affect brain mechanisms responsible for processing of auditory, visual and somatosensory modalities. Addiction to social networks was characterized by emotion regulation deficits reflected by reduced striatal activation during self-reflection compared to during ideal reflection in IGD players (63). This is an experimental manipulation of self-reflection which is related to brain activation and possibly can imply how the two interact. In conclusion, several studies have shown a consistent pattern of brain activation in response to video playing stimuli that is similar to activation of drug cues. Regions consistently activated by cue-exposure were the caudate nucleus, OCF, dorsolateral prefrontal cortex, inferior frontal cortex, anterior cingulate, PCC, para-hippocampus, and the precuneus (12). A single study (62) found an association between parts of the striatum and duration of IGD indicating long-term changes as result of play. These studies show how cue exposure can affect the brain’s reward, processing of sensory information and self-reflection.

### Inhibitory Control Mechanisms

Individuals with IGD display faulty inhibitory control mechanism such as impaired response inhibition on the Stroop task and related activity in the anterior and PCC (64). IGD participants also committed more commission errors on Go/No Go tasks and impaired response inhibition under gaming cue distraction (65). Impulsivity and response inhibition were associated with impaired function in the insula and greater activation of the frontal–striatal network in IGD (66). IGD participants also showed greater impulsivity and lower activity of motor areas while performing the Go/No Go task (67). In adolescents with IGD, there was increased activity in attention, and motor areas during No-Go trials (68). IGD participants failed to recruit frontal–basal ganglia pathway and inhibit unwanted actions on the Go-Stop paradigm (69). Furthermore, IGD participants showed higher activations when processing Internet gaming-related stimuli on a modified Stroop task in brain areas that are involved in selective attention, visual processing, working memory, and cognitive control (70).

### Recent Studies in IGD

A recent study found decreased left middle and superior temporal gyrus activation during interference of socially anxious words in IGD, possibly indicating social anxiety (71). A meta-analysis concluded that individuals with IGD are more likely to exhibit impaired response inhibition (72). In conclusion, these are consistent findings that the impairment in performance of response inhibition tasks is followed by failure to recruit frontal–basal ganglia pathways and use of other brain areas during inhibition in both adolescents and adults with IGD.

### Reward

Internet gaming disorder is associated with faulty decision-making and preference for immediate reward to long-term gains. IGD individuals subjectively experienced monetary gain and loss during the performance of a guessing task (73). IGD participants also showed increased activation in OCF in gain trials and decreased activation in the ACC during loss trials implicating enhanced reward sensitivity and decreased loss sensitivity. IGD participants also showed increased brain activity in other regions (the inferior frontal cortex, insula, ACC) and decreased activation in the caudate and PCC after continuous wins during performance on a continuous wins-and-losses task in fMRI (74). Finally, IGD participants preferred the probabilistic options to fixed ones and were faster to respond compared with control participants while performing on a probability-discounting task in fMRI (75). They also showed decreased activation in the inferior frontal gyrus and the precentral gyrus when choosing the probabilistic options than control participants. IGD participants also showed selection of risk-disadvantageous choices, and they make risky decisions more hastily and with less recruitment of regions implicated in impulse control (76). IGD adolescents had decreased reward sensitivity and they have been only sensitive to error monitoring regardless of positive feelings, such as sense of satisfaction (77). These finding imply impaired decision-making together with enhanced compensatory brain mechanisms that are consistent with impulsive decision-making.

### Recent Studies in IGD Participants

A recent study showed that negative outcomes affected the covariance between risk level and activation of brain regions related to value estimation (prefrontal cortex), anticipation of rewards (Ventral Striatum), and emotional-related learning (hippocampus) which may be one of the underlying neural mechanisms of disadvantageous risky decision-making in adolescents with IGD (78). IGD participants exhibited stronger functional connectivity when selecting small and immediate gains on a delay-discounting task (79). The results indicated that IGD participants have enhanced sensitivity to reward and decreased ability to control their impulsivity effectively, which leads to suboptimal decision-making (79). Males with IGD showed decision-making deficits indicating an imbalance between hypersensitivity for reward and weaker risk experience and self-control for loss (62). A recent review has suggested that both patients with IGD and those with pathological gambling exhibit decreased loss sensitivity; enhanced reactivity to gaming and gambling cues, enhanced impulsive choice behavior aberrant reward-based learning; and no changes in cognitive flexibility (80). In conclusion, IGD adolescents showed disadvantaged increased risk-taking choices and impaired ability to control their impulses similar to other impulse control disorders. The advantage of these studies is the use of simulated decision-making tasks to assess the effects of faulty decision-making processes on brain mechanisms responsible for reward.

## Brain Imaging Studies on Dopamine, 5-HT and Comorbid Psychiatric Disorders

Neurotransmitters such as DA, serotonin (5-HT) play an important role in drug and alcohol dependence, mainly by mediating dopamine reward and withdrawal mechanisms (81, 82). Consistent with evidence in drug and AUDs which are associated with deficient dopamine reward activity (83–86) IGD participants showed reduced levels of dopamine D_2_ receptor availability in the striatum (87) and reduced striatal dopamine transporter (DAT) availability (88). Finally, male IGD participants showed a significant decrease in glucose metabolism in the prefrontal, temporal, and limbic regions and lower levels of D_2_ receptor availability in the striatum (89). The results indicate that D_2_ receptor-mediated dysregulation of the OCF could underlie a mechanism for loss of control and compulsive behavior in IGD. Since there is no baseline measure of dopamine levels before the addiction it is not possible to determine whether dopamine deficiency is a predisposing factor for drug and AUD disorders or IGD. Magnetic resonance spectroscopy studies showed lower levels of N-acetylaspartate in the right frontal cortex and of choline in the medial temporal cortex in IGD participants that are similar to those of patients with attention deficit hyperactivity disorder (ADHD) and clinical depression (90). The studies so far support the evidence for deficient dopaminergic reward activity that classifies IGD as a behavioral addiction. The association between IGD and impaired self-regulation is also compatible with the model of IGD as an impulse control disorder lying within the impulsive–compulsive spectrum (1).

### Recent Studies on Comorbidity of IGD with ADHD and Depression

A recent study found that individuals with IGD showed altered PCC functional connectivity that might be dependent upon history of childhood ADHD (91). These findings suggest that altered neural networks for executive control in ADHD would be a predisposition for developing IGD. Furthermore, a study that used qualitative EGG to compare adolescents with IGD with or without ADHD found that Adolescents who show greater vulnerability to ADHD seem to continuously play Internet games to enhance attentional ability (92). Second, repetitive activation of brain reward and working memory systems during continuous gaming may result in an increase in neuronal connectivity within the parieto-occipital and temporal regions for the comorbid ADHD and IGD participants (92). Finally, a study that investigated the comorbidity of IGD with depression found that IGD participants with comorbid major depressive disorder (MDD) who performed on the Wisconsin card sorting task showed failure to suppress activity in the hippocampus during an attention demanding task, possibly as a consequence of depression (93). Patients with IGD have also shown an increased functional connectivity of several executive control brain regions that may relate to psychiatric comorbidity with ADHD and depression (94). Comorbidity of IGD with MDD was also indicated by decreased inter-hemispheric connectivity in the frontal region and vulnerability to attention problems in a study that used qualitative EEG (95). Furthermore, increased intrahemisphere connectivity in the fronto-temporo-parieto-occipital areas may result from excessive online gaming. The comorbidity with depression and ADHD may also associated with dopamine deficiency in IGD. Further studies need to investigate the similarity in neurochemical and neurocognitive brain circuits in IGD and comorbid conditions such as ADHD and depression.

## Discussion

The studies reviewed so far show consistent findings demonstrating the resemblance between the neural mechanisms underlying substance use disorder and IGD. The behavioral addiction model argues that IGD shows the features of excessive use despite adverse consequences, withdrawal phenomena, and tolerance that characterize substance use disorders. The evidence supports the behavioral addiction model of IGD by showing structural and functional changes in the mechanisms of reward and craving (but not withdrawal) in IGD. A recent meta-analysis found a significant activation of brain regions that mediate reward (the bilateral medial frontal gyrus and the left cingulate gyrus) in IGD (96). These studies support the notion that IGD is associated with changes to the brain’s reward system and mechanisms of loss of control and inhibition. There is also longitudinal evidence that pharmacological treatment with medication such as bupropion can attenuate cue reactivity in IGD (97) similar to the attenuation that occurs in nicotine-dependent users (98). IGD is associated with reduced brain’s DAT density and lower dopamine D_2_ receptor occupancy. It seems that excessive use of the brain’s dopamine reward system resembles the downregulation seen in case of drug and alcohol abuse, although in both disorder there are no baseline measures prior to the addiction precluding any inferences about causality. Finally, there is pharmacogenetic evidence that dopaminergic genes (Taq1A1 variation of dopamine D_2_ receptor and low activity Val158Met in the catecholamine-O-methyltransferase alleles) (99) and serotonergic genes (SS-5HTTLPR) together with personality factors may play a role in the vulnerability to IGD (100). The evidence for genetic dopaminergic vulnerability is compatible with the behavioral addiction model of IGD and consequently, IGD may be classified as a reward deficiency syndrome (101, 102). The evidence of genetic serotonergic vulnerability and brain imaging studies support the evidence of comorbidity of IGD with anxiety OCD and depression. Finally, playing games may be actually good for you and recent studies showed that playing computer game could improve the brain’s plasticity and thus be advantageous to certain conditions such as posttraumatic stress disorder, schizophrenia, and neurodegenerative disease (103).

One of the major limitations in brain imaging studies of IGD is they are mainly cross-sectional studies without baseline measures that rely on associations between structural and functional brain changes in the brain and Internet and videogame characteristics. These associations do not provide any proof that IGD activity plays a causal role in the development of the adolescent or adult brain. There are factors that may mediate such associations such as educational, cognitive, emotional and social factors. There are methodological considerations of age (use of adolescents and students), culture (most studies were done in the Far East), and lack of comparison groups with substance use disorders and these are major limitations of the studies that were reviewed so far. Finally, very few studies looked at sex differences in cognitive and brain function in IGD.

## Conclusion

There is an emerging evidence that IGD is associated with similar brain mechanisms responsible for substance use disorders. The brain imaging studies in IGD show similarity in brain mechanisms between IGD and substance use disorder and therefore support the classification of IGD as a behavioral addiction.

## Author Contributions

AW contributed substantially to the conception and design of the review.

## Conflict of Interest Statement

The author declares that this research was conducted in the absence of any commercial or financial relationships that could be construed as a potential conflict of interest.
